# The NLRP3 inflammasome: an emerging therapeutic target for chronic pain

**DOI:** 10.1186/s12974-021-02131-0

**Published:** 2021-03-30

**Authors:** Ruixiang Chen, Chengyu Yin, Jianqiao Fang, Boyi Liu

**Affiliations:** Department of Neurobiology and Acupuncture Research, The Third Clinical Medical College, Zhejiang Chinese Medical University, Key Laboratory of Acupuncture and Neurology of Zhejiang Province, 548 Binwen Road, Hangzhou, 310053 China

**Keywords:** NLRP3, Caspase-1, Inflammasome, Inflammation, Interleukins, Pain

## Abstract

Chronic pain affects the life quality of the suffering patients and posts heavy problems to the health care system. Conventional medications are usually insufficient for chronic pain management and oftentimes results in many adverse effects. The NLRP3 inflammasome controls the processing of proinflammatory cytokine interleukin 1β (IL-1β) and is implicated in a variety of disease conditions. Recently, growing number of evidence suggests that NLRP3 inflammasome is dysregulated under chronic pain condition and contributes to pathogenesis of chronic pain. This review provides an up-to-date summary of the recent findings of the involvement of NLRP3 inflammasome in chronic pain and discussed the expression and regulation of NLRP3 inflammasome-related signaling components in chronic pain conditions. This review also summarized the successful therapeutic approaches that target against NLRP3 inflammasome for chronic pain treatment.

## Introduction

Pain accompanies with many chronic diseases. Chronic pain due to tissue inflammation, nerve lesion, tumor invasion, or chemotherapy represents a major health problem in the health care system [1]. Chronic pain is among the most common complaints in outpatient clinic [2]. It is estimated that 11–40% of adult population suffers from chronic pain [1]. Moreover, chronic pain is usually accompanied with emotional changes, including anxiety, depression, or even suicidal tendencies [2]. Therefore, chronic pain dramatically affects the life quality and posts heavy economic and social burdens to the suffering patients. However, conventional treatments for chronic pain are still limited to nonsteroidal anti-inflammatory drugs (NSAIDs), opioids, corticosteroids, antidepressants, etc. These medications are usually insufficient for relieving chronic pain and often bring in many severe side effects [2, 3].

A large body of evidence indicates that inflammatory mediators (e.g., proinflammatory cytokines) in local inflamed tissues, peripheral nerves, and spinal cord make important contributions to the initiation and maintenance of chronic pain [3–5]. Among these proinflammatory cytokines, IL-1β is the most extensively studied cytokines. It exerts robust proinflammatory effects on many types of immune cells and tissues, whereas its excessive production is implicated in the pathophysiology of acute or chronic inflammation and pain. IL-1β may contribute to pain via direct and indirect mechanisms. On the one hand, IL-1β directly activates nociceptors to elicit action potentials and induce pain [6]. On the other hand, IL-1β contributes to peripheral or central sensitization by sensitizing nociceptors or promoting neuron-glia crosstalk [7–9]. Inhibition of IL-1β signaling has been shown to be effective for ameliorating pain in both animal models and human patients. Due to its important physiological function, the production of the active form of IL-1β is usually under tight regulation. One of the important mechanisms underlying such regulation is mediated via NLRP3 inflammasome.

## NLRP3 inflammasome and its activation mechanism

**Fig. 1:**
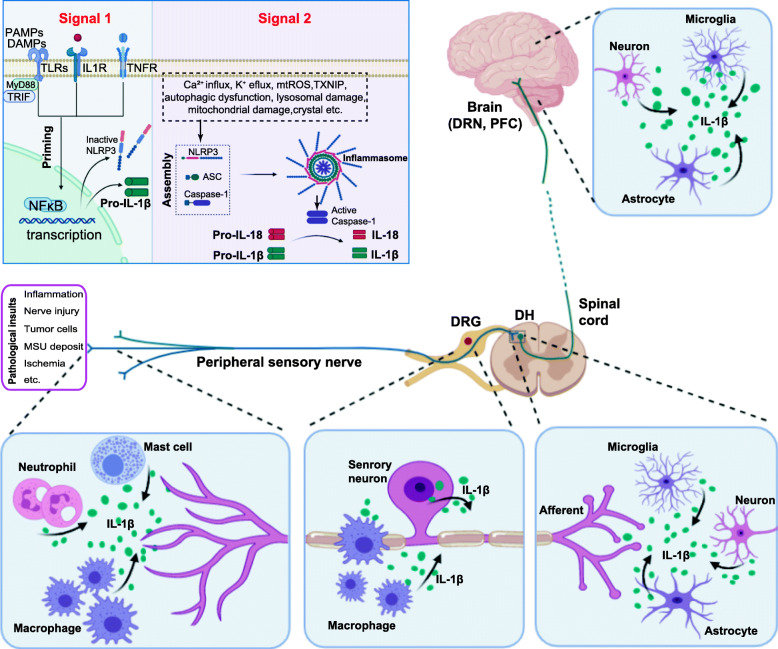
Schematic picture showing NLRP3 inflammasome activation and expression in the sensory nerve system and its surrounding microenvironment during chronic pain. The inset box depicts NLRP3 inflammasome activation procedure. The first phase (priming) involves Toll-like receptors (TLRs) and cytokine receptor recognition of the PAMPs/ DAMPs or endogenous cytokines, which results in activation of nuclear factor kappa B (NF-κB) signaling in the nucleus and upregulation of the transcription levels of inactive NLRP3 and pro-IL-1β. The second phase (assembly) involves the assembly of NLRP3 with ASC into inflammasome complex, initiated by the stimulation of NLRP3 by a plethora of stimuli, and subsequent activation of pro-caspase-1 with autocatalytic activity. The active caspase-1 ultimately cleaves pro-IL-1β and pro-IL-18, leading to maturation and release of IL-1β and IL-18. The expression of NLRP3 inflammasome has been identified in the following types of cells related with the sensory nerve system and surrounding microenvironment during chronic pain: peripheral inflamed tissues (including macrophages, neutrophils, mast cells) [14, 18, 19]; peripheral nerves and DRG (including DRG neurons and infiltrated macrophages) [20, 21]; spinal cord dorsal horn (including neurons, astrocytes and microglia) [15, 22]; brain regions (including neurons and astrocytes in DRN and microglia in PFC [23, 24]. This figure was created with BioRender.com

The NLRP3 inflammasome consists of NLRP3, ASC adaptors, and caspase-1 enzymes [10, 11]. The NLRP3 inflammasome is present primarily in immune and inflammatory cells, including mast cells, neutrophils, and macrophages, following activation by inflammatory stimuli [12–14]. Recent studies also identified NLRP3 inflammasome in neurons of the sensory nerve system [15]. NLRP3 inflammasome can be activated by a variety of stimuli and ligands, including PAMPs (pathogen associated molecular patterns), such as exogenous microbial molecules and bacterial lipopolysaccharide (LPS), and DAMPs (damage associated molecular patterns), such as HMGB1, S100 proteins, ATP, IL-33, and monosodium urate (MSU) [16, 17]. The activation procedure of NLRP3 inflammasome usually involves two phases (Fig. 1). The first phase is the priming phase, mediated primarily by Toll-like receptors (TLRs) and cytokine receptors (e.g., tumor necrosis factor receptor (TNFR)), which recognize PAMPs, DAMPs, or endogenous cytokines. This process results in upregulation of inactive NLRP3 and pro-IL-1β transcription via nuclear factor kappa B (NF-κB)-mediated transcriptional regulation [25]. MyD88 and TRIF, two downstream adaptor molecules of TLRs, regulate the induction of NLRP3 and pro-IL-1β transcription in response to TLR ligands during priming phase. The second phase involves the assembly of NLRP3 with ASC into the inflammasome complex, initiated by the stimulation of NLRP3 by a plethora of stimuli, including Ca^+^ influx, K^+^ efflux, mitochondrial damage, and ATP, and subsequent activation of pro-caspase-1 with autocatalytic activity (Fig. 1) [25]. The active caspase-1 ultimately cleaves pro-IL-1β and pro-IL-18, leading to maturation and release of IL-1β and IL-18 with proinflammatory activities [25]. The dysregulation of NLRP3 inflammasome has been shown to be related with a variety of diseases, including multiple sclerosis, diabetes, atherosclerosis, Alzheimer’s disease, inflammatory bowel disease, and many other autoimmune diseases [26]. More recently, growing number of evidence suggests that NLRP3 inflammasome is dysregulated under chronic pain conditions and contributes to the pathogenesis of chronic pain [27].

**Fig. 2:**
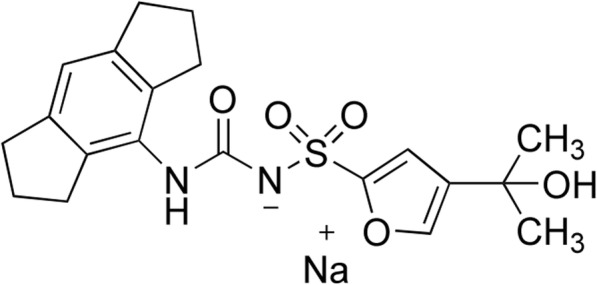
The chemical structure of the specific NLRP3 inflammasome antagonist MCC950. MCC950, a sulfonylurea molecule, was first discovered by Matthew Cooper et al., with original name CRID3, and then renamed MCC950 for “Matthew Cooper compound 950” [28]. MCC950 is a potent, selective, and small-molecule inhibitor of NLRP3 working at nM concentration. It specifically inhibited NLRP3 activation but not other inflammasome activation, including NLRP1, NLRC4, or AIM. Recent evidence suggests that the mechanism of MCC950 involves the closing of the open conformation of active NLRP3 [29].

Here in this review, we provide an up-to-date review of the recent findings of NLRP3 inflammasome in chronic pain conditions, including inflammatory pain, neuropathic pain, migraine, bone cancer pain, and morphine-induced analgesic tolerance and hyperalgesia. We discussed the expression and regulation of NLRP3 inflammasome-related genes and proteins during chronic pain. Furthermore, we also discussed the mechanisms underlying NLRP3 inflammasome’s contribution to chronic pain. Finally, this review summarized the successful therapeutic approaches that target against NLRP3 inflammasome for chronic pain, including the specific NLRP3 inflammasome antagonist MCC950 (Fig. 2, Table 2) [28] and other approaches (e.g., specific gene knockdown/knockout, electroacupuncture treatment).

## NLRP3 inflammasome in chronic pain

### NLRP3 in neuropathic pain

Neuropathic pain is one of the most intractable pain conditions and usually caused by injuries, lesions, or dysfunctions of the peripheral or central nervous system [36]. Nerve injuries can result in neuron damage, apoptosis, or death that release an array of DAMPs (e.g., HMGB1, IL-33, and ATP) to initiate NLRP3 inflammasome activation [37, 38]. Neuropathic pain results in a great decline of the life quality of patients, both physically and mentally. Up to date, there are a number of studies showing that NLRP3 is involved in neuropathic pain conditions, which are listed as follows:

#### NLRP3 in nerve injury-induced neuropathic pain

A study using partial sciatic nerve ligation (pSNL) model showed that pSNL injury increased the protein expression of NLRP3 inflammasome components, including NLRP3, ASC, caspase-1, and increased IL-1β in the spinal cord [39]. The authors found that NLRP3 inflammasome was a direct downstream effector of chemokine receptor CXCR4 in spinal cord glial cells, which was implicated in neuropathic pain [39]. Similar studies using chronic constriction injury (CCI)-induced neuropathic pain model also found that the expression of NLRP3 and its downstream effectors were increased in neurons and glia cells of spinal cord dorsal horn (Table 1) [42, 43]. Blocking connexin 43 hemichannels results in a significant improvement in mechanical pain hypersensitivity, accompanied with reductions in NLRP3, ASC, and caspase-1 protein expression in spinal cord [43].

**Table 1:** Expression of NLRP3 inflammasome components under different pain conditions

Tissue type	Pain condition	Marker	Cell type	Colocalized with	References
DRG	Bortezomib-induced neuropathic pain	Neun	Neuron	NLRP3	[21]
		IB4	Non-peptidergic neuron	NLRP3	
		NF200	Large diameter neuron	NLRP3	
		ED1	Macrophage	NLRP3	
	Paclitaxel-induced neuropathic pain	CD68	Macrophage	NLRP3	[20]
Sciatic nerve	Paclitaxel-induced neuropathic pain	CD68	Macrophage	NLRP3	[20]
Skin	CFA-induced inflammatory pain	CD68	Macrophage	NLRP3	[40]
		Cytokeratin	Keratinocyte	NLRP3	
		TCRα/β	T cell	NLRP3	
Muscle	Muscle pain	CD68	Macrophage	NLRP3	[41]
Spinal cord	CCI	Neun	Neuron	NLRP3	[42]
		GFAP	Astrocyte	NLRP3	
		CD11b	Microglia	NLRP3	
	Bone cancer pain	Neun	Neuron	NLRP3 ASC Caspase-1	[15]
		GFAP	Astrocyte	NLRP3 Caspase-1	
		Iba1	Microglia	NLRP3	
	CPIP	P2Y12	Microglia	IL-1β	[8]
	Morphine-induced hyperalgesia	Iba1	Microglia	NLRP3	[22]
TNC	Nitroglycerin-induced migraine	Iba1	Microglia	NLRP3 IL-1β	[34]
DRN	Morphine- or fentanyl-induced hyperalgesia	Neun	Neuron	NLRP3	[24]
		GFAP	Astrocyte	NLRP3	
PFC	Morphine analgesic tolerance	CD68	Microglia	ASC	[23]

Another study by Xu et al. found that overexpressing miR-34c improved CCI-induced neuropathic pain and spinal cord infarction and further alleviated cell apoptotic and inflammatory cytokine expression in CCI model mice [42]. The therapeutic effect of miR-34c is accompanied with decreased protein levels of NLRP3, ASC, caspase-1, IL-1β, and IL-18 in the spinal cord of CCI mice. Bioinformatic analyses and biochemical assays further revealed that miR-34c suppressed the expression of NLRP3 by directly binding to the 3'-untranslated region. Further experiments that specifically target against NLRP3 inflammasome will be needed to further verify its role in nerve injury-induced neuropathic pain [42]. Therefore, these studies suggest that NLRP3 inflammasome may be potentially involved in nerve injury-induced neuropathic pain and inflammation.

#### NLRP3 in chemotherapy-induced peripheral neuropathy (CIPN)

CIPN is one of the most serious complications caused by anticancer drugs [44]. Neuropathic pain is a major clinical symptom accompanying CIPN, which includes tingling, burning pain, and numbness in the feet and hands. The sensory abnormalities and pain can even become chronic and persist after chemotherapy is terminated, which severely affect the life quality of the patients [45]. Neoplastic cancer cells and chemotherapeutic drugs can lead to the release of DAMPs by cancer cells per se or by host cells [37]. In a paclitaxel-induced peripheral neuropathic pain rat model, paclitaxel treatment resulted in elevated expression of NLRP3, caspase-1, and IL-1β in dorsal root ganglion (DRG) and sciatic nerve [20]. The expression of NLRP3 was primarily expressed in macrophages that infiltrated in DRG and sciatic nerve (Table 1). Paclitaxel also caused mitochondrial damage and production of reactive oxygen species (ROS) in sciatic nerve, which triggered the activation of NLRP3 inflammasome [20]. Application of phenyl-N-tert-butylnitrone (a ROS scavenger) significantly reduced paclitaxel-induced mechanical allodynia and inhibited NLRP3 inflammasome activation in DRG and sciatic nerve [20].

In another CIPN animal model induced by bortezomib, the expression of NLRP3 was significantly increased in DRG. Further immunostaining results showed that NLRP3 expression was primarily located in DRG neurons and infiltrating macrophages, but not in satellite glial cells (Table 1) [21]. Knockdown of *Nlrp3* gene expression via intrathecal siRNA injection significantly prevented bortezomib-induced mechanical allodynia. In contrast, the overexpression of *Nlrp3* gene in DRG significantly reduced the paw withdrawal threshold in treated animals [21].

In addition, in oxaliplatin-induced CIPN animal model, the expression of NLRP3 and caspase-1 were increased in spinal cord. Blocking NLRP3 activation by daily intrathecal injection of MCC950 significantly alleviated oxaliplatin-induced neuropathic pain (Table 2) [30]. In all, these results indicate NLRP3 inflammasome plays an important role in CIPN-induced neuropathic pain.

**Table 2:** Application of MCC950 for treating different pain conditions

Pain condition	Species	Administration route	Administration dosage	Dosage regimen	References
Oxaliplatin-induced CIPN	SD rats	i.t.	5 μmol/day	Once daily for 25 consecutive days	[30]
CPIP	SD rats	i.t.	30 μg/rat	Once daily for 7 consecutive days	[31]
RR-EAE	C57 mice	i.g.	50 mg/kg	Once daily for 21 consecutive days	[32]
FSL1-induced inflammatory pain	C57 mice	i.p.	10 mg/kg	Single dose	[33]
NTG-induced migraine	C57 mice	i.p.	10 mg/kg	Once daily for 11 consecutive days	[34]
EAP	NOD/LtJ non-obese diabetic (NOD) mice	i.p.	10 mg/kg	Once a day or every other day for a total of 7 treatments	[35]
BCP	SD rats	i.p.	5 mg/kg; 10 mg/kg	For acute treatment: single dose	[15]
			5 mg/kg; 10 mg/kg	For chronic treatment: once daily for 5 consecutive days	
Morphine- or fentanyl-induced hyperalgesia	Wistar rats	i.p.	5 mg/kg	Once daily for 7 consecutive days	[24]

#### NLRP3 in complex regional pain syndrome type-I (CRPS-I)

CRPS-I is a progressive and devastating neuropathic pain condition that usually affects the limbs and is not accompanied with a clinically verifiable nerve injury [46]. CRPS-I usually develops after an initial injury, including ischemia, trauma, surgery, or fractures to the extremity. The pain is out of proportion to the severity of the initial injury and becomes chronic. CRPS patients usually suffer from excruciating and chronic pain in affected areas, leading even to disabilities. Unfortunately, no specific drugs haven been approved for CRPS-I treatment at present. Conventional medications, including NSAIDs, corticosteroids, and narcotics usually do not produce satisfactory relieving effects on CRPS-I, making it a challenging pain condition in clinic [47, 48]. To explore the mechanisms underlying CRPS-I, several preclinical animal models, including the rat chronic post-ischemic pain (CPIP) model, have been developed to recapitulate CRPS-I [49, 50].

To further explore the mechanisms underlying CRPS-I, our recent study performed transcriptome profiling of gene expression changes in ipsilateral DRG and spinal cord dorsal horn (SCDH) of CPIP model rats by means of RNA-Sequencing [31, 51, 52]. We identified *Nlrp3* gene expression to be significantly upregulated in SCDH of the CPIP model rats. We further identified that the gene and protein expressions of NLRP3, caspase-1, and IL-1β were all significantly increased in ipsilateral SCDH of CPIP model rats. Pharmacological blockade of NLRP3 by intrathecal delivery of the specific NLRP3 antagonist MCC950 reduced IL-1β overproduction and glial activation in ipsilateral SCDH as well as mechanical allodynia of CPIP model rats (Table 2) [31]. It is known that spinal IL-1β and glia cell activation mediate pain mechanisms of CRPS-I (Table 1) [8, 53, 54]. Therefore, NLRP3 inflammasome is activated in SCDH of a rat model of CRPS-I and it contributes to the mechanical allodynia via promoting IL-1β overproduction and glia cell activation in SCDH. This study indicates that NLRP3 inflammasome may be a novel therapeutic target for CRPS-I management.

#### NLRP3 in central neuropathic pain (CNP)

CNP is common among patients with neurological complications of the central nervous system insult, such as spinal cord injury, stroke, or multiple sclerosis [55]. However, at present, the provision of effective pain alleviations to CNP can be difficult due to many unwanted side effects of conventional medications. Recently, Khan and colleagues established an optimized relapsing-remitting experimental encephalomyelitis (RR-EAE) mouse model to mimic human multiple sclerosis [32]. The RR-EAE model mice developed obvious mechanical allodynia in bilateral hind limbs. Daily oral administration of the NLRP3 antagonist MCC950 progressively and effectively alleviated the established mechanical allodynia in bilateral hind limbs of model mice (Table 2) [32]. Furthermore, MCC950 also improved the disease relapses in RR-EAE model mice, indicated by the improvement of hind limb weakness and tail limpness [32]. These results indicated that targeting NLRP3 inflammasome could be a potential method for alleviating multiple sclerosis-induced CNP and disease relapses.

In the mouse model of spinal cord injury (SCI), significant locomotor dysfunction and mechanical/thermal hyperalgesia were developed, accompanied with NLRP3 inflammasome activation and production of IL-1β and IL-18 in spinal cord. Treatment with D-β-hydroxybutyrate (DBHB), an endogenous ketone body NLRP3 inflammasome inhibitor, markedly improved locomotor function and relieved SCI-induced pain hypersensitivities, accompanied with attenuated NLRP3 inflammasome activation and protein expression of IL-1β and IL-18 in the spinal cord [56, 57], demonstrating a possible involvement of NLRP3 inflammasome in SCI-induced CNP.

### NLRP3 in inflammatory pain

Inflammatory pain is usually triggered by infection, trauma, or tissue damage, which induces the release of an array of inflammatory mediators (e.g., bradykinin, prostaglandin, H^+^, ATP, nerve growth factor, endothelin), proinflammatory cytokines, chemokines, etc. [3, 58–60]. PAMPs (e.g., LPS) and DAMPs (e.g., MSU, S100 proteins) may be both involved in inflammatory pain, depending on specific inflammatory conditions [37, 61, 62]. The inflammatory mediators, cytokines, or chemokines can directly stimulate or sensitize the nociceptors in surrounding tissues and mediate inflammatory pain [3, 4].

#### NLRP3 in complete Freund’s adjuvant (CFA)-induced inflammatory pain

In CFA-induced animal inflammatory pain model, the expression of NLRP3 inflammasome components, including ASC, caspase-1, and IL-1β, are significantly increased in local inflamed hind paw tissues and spinal cord [40, 63, 64]. Immunostaining revealed that NLRP3 was colocalized with markers for keratinocytes, T cells, and macrophages (Table 1). Repetitive electroacupuncture (EA) treatment produced analgesic effects on CFA-induced pain model mice, accompanied with reduced NLRP3 inflammasome activation and IL-1β production in local inflamed tissues and especially in macrophages [40, 63]. Since IL-1β is known to be an important contributor to CFA-induced inflammatory pain, these results suggest that EA-induced analgesic effects may possibly be related with its inhibitory effects on NLRP3 inflammasome activation in local inflamed tissues [7]. Experiments that specifically target against NLRP3, e.g., using specific antagonist or gene knockdown approaches, will be further needed to elucidate the exact contributions of NLRP3 inflammasome in CFA-induced inflammatory pain condition.

Recently, another study by Huang et al. reported that the expression of TLR2 and TLR6 were significantly upregulated in plantar skin of CFA-induced inflammatory pain model. Selective activation of TLR2/TLR6 by agonist FSL1 triggered acute inflammatory pain, as well as a significant increase in *Nlrp3* gene expression in hind paw tissues [33]. They found that the acute inflammatory pain was significantly reduced in *Nlrp3* gene knockout animals or by MCC950 treatment (Table 2). Further evidence showed that the pain response was mediated via mechanisms involving NLRP3-dependent upregulation of IL-33, a pain-producing cytokine known to be involved in many pain conditions [65, 66]. Therefore, this study revealed an important role of NLRP3-dependent upregulation of IL-33 in mediating inflammatory pain.

#### NLRP3 in monosodium urate crystal-induced gout pain

Gout is evoked by monosodium urate (MSU) crystal deposition in joint and periarticular tissues. The deposition of MSU crystals triggers the activation of innate immune system, including neutrophil and macrophage influx, which induces intense inflammation and pain in local joint and periarticular tissues. Gout pain is the most common type of inflammatory arthritis and dramatically affects the life quality of patients [67]. Unfortunately, the incidence of gout is still constantly rising due to the aging population and dietary changes [68].

Mounting evidence indicates that MSU-induced inflammation and pain response depend on IL-1β release [69, 70]. Blocking IL-1 signaling with either recombinant IL-1 receptor antagonist or neutralizing antibodies of IL-1 or IL-1 receptor attenuates neutrophil infiltration in response to MSU challenge [71, 72]. In accordance, mice deficient in IL-1 receptor showed much reduced ankle edema and pain response after MSU injection into ankle joint [73]. MSU stimulates macrophages to release IL-1β via NLRP3 inflammasome activation. In a mouse model of MSU-induced peritonitis, macrophages from mice lacking the key components of NLRP3 inflammasome, e.g., NLRP3, caspase-1, or ASC, were incapable of producing the active form of IL-1β in response to MSU challenge [18]. Furthermore, in a mouse gout model by intraarticular MSU injection, mice deficient in NLRP3, caspase-1, or ASC showed much reduced pain response as well as reduced neutrophil infiltration and IL-1β in ankle joints [74]. In addition to macrophages, IL-1β production from other cellular sources should not be neglected. For example, IL-1β can be released from mast cells by activation of NLRP3 inflammasome [13]. Mast cell-derived IL-1β made important contributions to MSU-induced ankle inflammation, especially during early phase [19].

Despite much progress has been made in elucidating the importance of NLRP3 inflammasome in gout pathology, the precise mechanisms through which NLRP3 inflammasome is activated under gout condition remain elusive. Our recent work, together with others, demonstrated that the production of endogenous ROS are increased during gout condition and are critically involved in mediating gout pain and inflammation [66, 75–77]. It is known that the generation of ROS induces dissociation of ROS-sensitive NLRP3 ligand thioredoxin-interacting protein (TXNIP) from its inhibitor thioredoxin (TRX). TXNIP then binds to NLRP3 and leads to NLRP3 inflammasome activation [78, 79]. We further found that eliminating ROS productions by applying the natural product eucalyptol or classical antioxidants largely reduced NLRP3 inflammasome activation in vivo, which results in less caspase-1 and IL-1β expression in local inflamed ankle tissues and gout pain relief in a mouse MSU-induced gout model [75]. These results suggest that ROS are important triggers for NLRP3 inflammasome activation under gout condition and eliminating ROS overproduction may represent promising therapeutic options for gout arthritis.

At present, several clinical studies have proven that therapies targeting against IL-1β are successful strategies for relieving pain, inflammation, and recurrent attacks of gout, indicating that targeting IL-1β and its related pathways is effective for gout treatment [69]. Therefore, targeting ROS, NLRP3 inflammasome, and its downstream signaling may offer promising therapeutic options for gout.

#### NLRP3 in postoperative pain

The development of chronic postoperative pain after surgery constitutes a major clinical problem [80]. Postoperative pain possesses both inflammatory and neuropathic pain properties, which arise from tissue damage and incision of nerve endings at the surgery site. Studies have shown that IL-1β is significantly increased at the incision site [81, 82]. Inhibiting IL-1β signaling either by receptor antagonist or deletion of receptor IL-1R1 alleviated postoperative pain in both mouse model and human patients, suggesting IL-1β is a key contributor to postoperative pain [83–86]. Many endogenous activators of NLRP3 inflammasome, including ROS, ATP, and DAMPs, are released due to tissue damage after surgery, suggesting the possible activation of NLRP3 inflammasome during postoperative pain.

A recent study by Cowie et al. found that male mice deficient in NLRP3 showed less inflammation at the surgery site and recovered from surgery-induced pain faster than wild type controls. In contrast, female mice deficient in NLRP3 showed only modest attenuation of postoperative pain and no change in postoperative inflammation [82]. Further studies indicated that NLRP3 deletion reduced IL-1β expression only in males but not in females, suggesting that IL-1β production in females occurs via mechanisms independent of NLRP3 [82]. The authors continued to investigate this intriguing sex dimorphism by selectively deleting NLRP3 in peripheral sensory neurons. They found that NLRP3 expressed in non-neuronal cells and sensory neurons both contributed to the postoperative pain in male mice. In contrast, in female mice, the contribution of sensory neurons to postoperative pain is NLRP3 dependent, whereas the contribution of other non-neuronal cells is NLRP3 independent [82]. The exact immunological and neurological mechanisms underlying such drastic sex dimorphism of NLRP3’s contribution in postoperative pain is still unknown and awaiting further investigation [87]. But this study suggests targeting NLRP3 may be a novel strategy for alleviating postoperative pain and associated inflammation. More importantly, it also suggests that gender differences should be taken into consideration when evaluating therapeutic effects of NLRP3 antagonism on postoperative pain in preclinical animal models and further translational studies.

#### NLRP3 in muscle pain

Muscle pain is a very common type of pain condition and is usually induced by stress, tension, muscle overuse, or minor injuries [88]. Muscle pain can take place in a small area or even the whole body, ranging from mild to severe conditions [89]. Although most of muscle pains can recover on their own in a short period, it sometimes persists over months under certain pathological conditions (e.g., myofascial pain syndrome and fibromyalgia) and significantly affects life quality of the patients [90].

The role of NLRP3 inflammasome activation in a mouse model of muscle pain has recently been investigated by Yoshida et al. [41]. They established a mouse model of muscle pain using electrical stimulation to induce excessive muscle contraction in the mouse hind leg to mimic over exercise-induced muscle pain in humans. They observed that the stimulated hind leg developed obvious mechanical allodynia, accompanied with increased levels of uric acid, NLRP3, caspase-1 activity, IL-1β, and the number of macrophages, when compared with that of non-stimulated leg. Muscle overuse can trigger the increase of uric acid in the plasma [91]. When the level of uric acid in the plasma reaches its limit of solubility, MSU crystal formation then takes place and triggers NLRP3 inflammasome activation [18]. The authors continued to test the effects of caspase-1 inhibition, macrophage depletion, and blocking uric acid production using xanthine oxidase inhibitor. Administration of these reagents all reduced mechanical pain hypersensitivities in model mice, suggesting IL-1β released by NLRP3 inflammasome activation from macrophages contributed to mechanical pain hypersensitivity [91]. It should be noted that the electrical stimulation-induced muscle pain model may not completely recapitulate the pathophysiology underlying chronic muscle pain. This study suggests that pharmacological blocking NLRP3 inflammasome may be potentially utilized to alleviate muscle pain due to over-exercise in humans.

### NLRP3 in migraine

Migraine is the most common type of disabling primary headache worldwide, which is characterized with moderate to severe throbbing or pulsing pain sensation [92]. Migraine usually lasts for hours or even days, which significantly affects the daily activities of the suffering patients [92, 93]. It is found that the serum level of IL-1β is significantly increased among migraine patients compared with healthy controls and correlated positively with the level of calcitonin gene-related peptide (CGRP), a well-established neuropeptide implicated in migraine pathology [94–96]. Recently, He et al. established a mouse migraine model by repeated nitroglycerin (NTG) administration [34]. Repeated NTG application triggered both acute and persistent mechanical pain hypersensitivity in periorbital area, an indication of migraine-like behavior in mouse [97]. Furthermore, the authors found that NLRP3 and IL-1β expression was significantly upregulated in the microglia of trigeminal nucleus caudalis (TNC) (Table 1), a place that receives and integrates pain signals from the trigeminal area and is considered as a central area relevant for migraine [98]. Pharmacological blocking NLRP3 (Table 2) or IL-1β with the NLRP3 antagonist MCC950 or IL-1ra not only improved NTG-induced hyperalgesia, but also inhibited the biomarkers related to central sensitization of migraine in TNC, such as p-ERK, c-Fos, and CGRP. These results indicate that the activation of NLRP3 in TNC contributes to nitroglycerin (NTG)-induced migraine-like behavior via promoting neuroinflammation and central sensitization [34]. Thus, inhibition of NLRP3 inflammasome may represent a potential therapeutic approach for alleviating migraine.

### NLRP3 in visceral pain

Visceral pain mainly refers to pain originating from internal organs of the body (e.g., thoracic, abdominal, or pelvic organs). Stretching, inflammation, ischemia, pH, bacteria, immune mediators, and neurotransmitters can all evoke visceral pain. Therefore, PAMPs and DAMPs may both be involved in visceral pain. Among visceral pains, chronic pelvic pain syndrome (CPPS), also known as type III prostatitis [99], is the most prevalent urogenital disease among males with age < 50 years old. It causes pain and inflammation in the prostate, lower urinary tract, and pelvic area [100]. Recently, Zhang and colleagues explored the role of NLRP3 inflammasome in the pathogenesis of CPPS [35]. They established a mouse model of experimental autoimmune prostatitis (EAP) to mimic CPPS via intradermal injection of a mixture of prostate antigens and CFA. The EAP model mice developed obvious mechanical allodynia in the lower abdominal area nearby prostate. In addition, NLRP3, caspase-1, ASC, and IL-1β were all upregulated in prostate tissue of EAP model mice. Epidemiological studies reported an association between alcohol consumption and CPPS [101]. Then the authors found that treating EAP model mice with alcohol further exacerbated the pain response and promoted NLRP3 inflammasome activation in EAP model mice. The treatment with MCC950 inhibited NLRP3 inflammasome activation and reduced the mechanical allodynia of EAP model mice (Table 2). Furthermore, MCC950 attenuated the aggravated severity of alcohol-treated EAP model mice [35]. Therefore, NLRP3 inflammasome may be a promising therapeutic target for relieving pelvic pain and inflammation associated with CPPS and especially CPPS with alcohol consumption.

### NLRP3 in cancer related pain

Pain is a common symptom among patients with cancer. It is reported that up to 75% of cancer patients suffer from severe bone pain induced by cancer [3]. Bone cancer pain (BCP) occurs in patients with primary bone cancer or with bone metastasis from other distal regions, such as prostate, lung, and breast cancer [102]. BCP can be debilitating and poses a heavy burden on patients’ daily activities and mental health [102, 103]. Mechanistically, BCP is considered as a specific type of pain condition with overlapping but distinct features of both inflammatory and neuropathic pain [102]. The tumor and surrounding microenvironment released a number of DMAPs (e.g., HMGB1, S100 proteins), which could trigger NLRP3 inflammasome activation [104]. Unfortunately, our current knowledge of the underlying mechanisms of BCP is still limited compared with the understandings of other inflammatory or neuropathic pain.

Recently, Chen and colleagues investigated the role of spinal NLRP3 inflammasome in the development of BCP [15]. A rat BCP model was established by inoculating Walker 256 carcinoma cells into the medullary cavity of the rat tibia. Mechanical allodynia and inflammasome activation were then evaluated, and it was found that BCP model rats developed mechanical allodynia, accompanied with increased expression of NLRP3 inflammasome, including NLRP3, ASC, caspase-1, and IL-1β, primarily in neurons of spinal cord dorsal horn (Table 1). MCC950 administration in BCP model rats reduced the upsurge of NLRP3 inflammasome and IL-1β protein expression in spinal cord dorsal horn and further relieved the mechanical allodynia (Table 2) [15]. These results suggest spinal NLRP3 inflammasome may be a novel target for BCP treatment.

### NLRP3 in opioid analgesic tolerance and hyperalgesia

Morphine and fentanyl are opioids widely used for relieving pain, but prolonged usage in patients with chronic pain can end up in analgesic tolerance and hyperalgesia. These signs are two major adverse effects of prolonged morphine or fentanyl treatment, which severely reduced their clinical usages [105]. Prolonged morphine treatment can induce significant NLRP3 inflammasome activation in spinal cord of an animal model of morphine-induced analgesic tolerance and hyperalgesia [106, 107]. Further evidence demonstrates morphine treatment can induce a persistent release of DMAPs (including HMGB1, biglycan, heat shock protein 90) in spinal cord [108]. The contribution of NLRP3 inflammasome to morphine-induced analgesic tolerance and hyperalgesia is further supported by a study from Grace et al., who found that the knockdown of spinal *Nlrp3* gene expression by siRNA resulted in alleviation of the prolongation of neuropathic pain induced by morphine treatment in a mouse CCI neuropathic pain model [22]. Mechanistically, they found that NLRP3 was primarily distributed in microglia of spinal cord dorsal horn (Table 1). Selective silencing spinal microglia via chemogenetic approaches prevented and persistently reversed morphine-induced prolongation of neuropathic pain [22].

In addition to spinal cord, recent studies identified significant activation of NLRP3 inflammasome in prefrontal cortex (PFC), dorsal raphe nucleus (DRN), and peripheral blood of morphine- or fentanyl-induced analgesic tolerance and hyperalgesia model animals [23, 24]. NLRP3 expression was primarily upregulated in astrocytes and serotonergic neurons in DRN following morphine or fentanyl treatment (Table 1). MCC950 delayed morphine and fentanyl analgesic tolerance and prevented fentanyl-induced hyperalgesia (Table 2) [24]. Besides, morphine-induced analgesic tolerance and hyperalgesia are abolished in *Nlrp3* gene knockout mice. Mechanistic studies further revealed that prolonged morphine treatment induced the production of ROS, which triggers NLRP3 inflammasome activation in microglia in PFC (Table 1) [23], suggesting a critical contribution of ROS-mediated NLRP3 inflammasome activation in microglia to the maintenance of morphine-induced analgesic tolerance and hyperalgesia. Therefore, counteracting ROS by antioxidants or with NLRP3 antagonist may provide novel targets for preventing the development of opioid analgesic tolerance and hyperalgesia.

## Conclusions

In this review, we summarized the recent advances in our understanding of the involvement of NLRP3 inflammasome in chronic pain. Recently, mounting evidence suggest that NLRP3 inflammasome is activated in local tissues, peripheral sensory nerves and neurons, spinal cord, and brain regions of a variety of animal models of chronic pain. These regions are all related with pain signal initiation, transduction, integration, and perception (Fig. 1). Pharmacological studies using NLRP3 inflammasome antagonist or genetic approaches further confirmed the participation of NLRP3 inflammasome and its downstream signaling components in mediating chronic pain. Therefore, it has become more and more accepted that NLRP3 inflammasome plays an important role in the pathogenesis of chronic pain. Further preclinical and translational studies will be needed to evaluate the safety and effectiveness of drugs or approaches that specifically target against NLRP3 inflammasome in chronic pain treatment.

## Data Availability

Not applicable.

## Abbreviations

IL-1β: Interleukin 1β
LPS: Lipopolysaccharide
NSAIDs: Nonsteroidal anti-inflammatory drugs
PAMPs: Pathogen associated molecular patterns
DAMPs: Damage-associated molecular patterns
DRG: Dorsal root ganglion
MSU: Monosodium urate
TLRs: Toll-like receptors
MyD88: Myeloid differentiation primary response 88
TNFR: Tumor necrosis factor receptor
NF-κB: Nuclear factor kappa B
pSNL: Partial sciatic nerve ligation
CCI: Chronic constriction injury
CIPN: Chemotherapy-induced peripheral neuropathy
CRPS-I: Complex regional pain syndrome type-I
CPIP: Chronic post-ischemic pain
SCDH: Spinal cord dorsal horn
CNP: Central neuropathic pain
SCI: Spinal cord injury
RR-EAE: Relapsing-remitting experimental encephalomyelitis
DBHB: D-β-hydroxybutyrate
CFA: Complete Freund’s adjuvant
EA: Electroacupuncture
ROS: Reactive oxygen species
TXNIP: Thioredoxin-interacting protein
TRX: Thioredoxin
CGRP: Calcitonin gene-related peptide
NTG: Nitroglycerin
TNC: Trigeminal nucleus caudalis
CPPS: Chronic pelvic pain syndrome
EAP: Experimental autoimmune prostatitis
BCP: Bone cancer pain
DRN: Dorsal raphe nucleus
PFC: Prefrontal cortex

## References

[1] Dahlhamer J, Lucas J, Zelaya C, Nahin R, Mackey S, DeBar L, Kerns R, Von Korff M, Porter L, Helmick C (2018). Prevalence of chronic pain and high-impact chronic pain among adults - United States, 2016. MMWR Morb Mortal Wkly Rep.

[2] Dydyk AM, Conermann T (2020). Chronic pain.

[3] Jiang BC, Liu T, Gao YJ (2020). Chemokines in chronic pain: cellular and molecular mechanisms and therapeutic potential. Pharmacol Ther.

[4] Ji RR, Xu ZZ, Gao YJ (2014). Emerging targets in neuroinflammation-driven chronic pain. Nat Rev Drug Discov.

[5] Ji RR, Chamessian A, Zhang YQ (2016). Pain regulation by non-neuronal cells and inflammation. Science.

[6] Binshtok AM, Wang H, Zimmermann K, Amaya F, Vardeh D, Shi L, Brenner GJ, Ji RR, Bean BP, Woolf CJ, Samad TA (2008). Nociceptors are interleukin-1beta sensors. J Neurosci.

[7] Safieh-Garabedian B, Poole S, Allchorne A, Winter J, Woolf CJ (1995). Contribution of interleukin-1 beta to the inflammation-induced increase in nerve growth factor levels and inflammatory hyperalgesia. Br J Pharmacol.

[8] Helyes Z, Tekus V, Szentes N, Pohoczky K, Botz B, Kiss T, Kemeny A, Kornyei Z, Toth K, Lenart N (2019). Transfer of complex regional pain syndrome to mice via human autoantibodies is mediated by interleukin-1-induced mechanisms. Proc Natl Acad Sci U S A.

[9] Li T, Chen X, Zhang C, Zhang Y, Yao W (2019). An update on reactive astrocytes in chronic pain. J Neuroinflammation.

[10] Alexander SPH, Kelly E, Mathie A, Peters JA, Veale EL, Armstrong JF, Faccenda E, Harding SD, Pawson AJ, Sharman JL (2019). THE CONCISE GUIDE TO PHARMACOLOGY 2019/20: Introduction and Other Protein Targets. Br J Pharmacol.

[11] O'Brien WT, Pham L, Symons GF, Monif M, Shultz SR, McDonald SJ (2020). The NLRP3 inflammasome in traumatic brain injury: potential as a biomarker and therapeutic target. J Neuroinflammation.

[12] Zhang T, Fang Z, Linghu KG, Liu J, Gan L, Lin L (2020). Small molecule-driven SIRT3-autophagy-mediated NLRP3 inflammasome inhibition ameliorates inflammatory crosstalk between macrophages and adipocytes. Br J Pharmacol.

[13] Nakamura Y, Kambe N, Saito M, Nishikomori R, Kim YG, Murakami M, Nunez G, Matsue H (2009). Mast cells mediate neutrophil recruitment and vascular leakage through the NLRP3 inflammasome in histamine-independent urticaria. J Exp Med.

[14] Goldberg EL, Asher JL, Molony RD, Shaw AC, Zeiss CJ, Wang C, Morozova-Roche LA, Herzog RI, Iwasaki A (2017). Dixit VD: beta-hydroxybutyrate deactivates neutrophil NLRP3 inflammasome to relieve gout flares. Cell Rep.

[15] Chen SP, Zhou YQ, Wang XM, Sun J, Cao F, HaiSam S, Ye DW, Tian YK (2019). Pharmacological inhibition of the NLRP3 inflammasome as a potential target for cancer-induced bone pain. Pharmacol Res.

[16] Roh JS, Sohn DH (2018). Damage-associated molecular patterns in inflammatory diseases. Immune Netw.

[17] Gong T, Yang Y, Jin T, Jiang W, Zhou R (2018). Orchestration of NLRP3 inflammasome activation by ion fluxes. Trends Immunol.

[18] Martinon F, Petrilli V, Mayor A, Tardivel A, Tschopp J (2006). Gout-associated uric acid crystals activate the NALP3 inflammasome. Nature.

[19] Reber LL, Marichal T, Sokolove J, Starkl P, Gaudenzio N, Iwakura Y, Karasuyama H, Schwartz LB, Robinson WH, Tsai M, Galli SJ (2014). Contribution of mast cell-derived interleukin-1beta to uric acid crystal-induced acute arthritis in mice. Arthritis Rheumatol.

[20] Jia M, Wu C, Gao F, Xiang H, Sun N, Peng P, Li J, Yuan X, Li H, Meng X (2017). Activation of NLRP3 inflammasome in peripheral nerve contributes to paclitaxel-induced neuropathic pain. Mol Pain.

[21] Liu CC, Huang ZX, Li X, Shen KF, Liu M, Ouyang HD, Zhang SB, Ruan YT, Zhang XL, Wu SL, Xin WJ, Ma C (2018). Upregulation of NLRP3 via STAT3-dependent histone acetylation contributes to painful neuropathy induced by bortezomib. Exp Neurol.

[22] Grace PM, Strand KA, Galer EL, Urban DJ, Wang X, Baratta MV, Fabisiak TJ, Anderson ND, Cheng K, Greene LI, Berkelhammer D, Zhang Y, Ellis AL, Yin HH, Campeau S, Rice KC, Roth BL, Maier SF, Watkins LR (2016). Morphine paradoxically prolongs neuropathic pain in rats by amplifying spinal NLRP3 inflammasome activation. Proc Natl Acad Sci U S A.

[23] Liu Q, Su LY, Sun C, Jiao L, Miao Y, Xu M, Luo R, Zuo X, Zhou R, Zheng P, Xiong W, Xue T, Yao YG (2020). Melatonin alleviates morphine analgesic tolerance in mice by decreasing NLRP3 inflammasome activation. Redox Biol.

[24] Carranza-Aguilar CJ, Hernandez-Mendoza A, Mejias-Aponte C, Rice KC, Morales M, Gonzalez-Espinosa C, et al. Morphine and fentanyl repeated administration induces different levels of NLRP3-dependent pyroptosis in the dorsal raphe nucleus of male rats via cell-specific activation of TLR4 and opioid receptors. Cell Mol Neurobiol. 2020. 10.1007/s10571-020-00957-5. 10.1007/s10571-020-00957-5 PMC11441185 32926257

[25] He Y, Hara H, Nunez G (2016). Mechanism and regulation of NLRP3 inflammasome activation. Trends Biochem Sci.

[26] Shao BZ, Xu ZQ, Han BZ, Su DF, Liu C (2015). NLRP3 inflammasome and its inhibitors: a review. Front Pharmacol.

[27] Zhang H, Li F, Li WW, Stary C, Clark JD, Xu S, Xiong X (2016). The inflammasome as a target for pain therapy. Br J Anaesth.

[28] Coll RC, Robertson AA, Chae JJ, Higgins SC, Munoz-Planillo R, Inserra MC, Vetter I, Dungan LS, Monks BG, Stutz A (2015). A small-molecule inhibitor of the NLRP3 inflammasome for the treatment of inflammatory diseases. Nat Med.

[29] Tapia-Abellan A, Angosto-Bazarra D, Martinez-Banaclocha H, de Torre-Minguela C, Ceron-Carrasco JP, Perez-Sanchez H, Arostegui JI, Pelegrin P (2019). MCC950 closes the active conformation of NLRP3 to an inactive state. Nat Chem Biol.

[30] Wahlman C, Doyle TM, Little JW, Luongo L, Janes K, Chen Z, Esposito E, Tosh DK, Cuzzocrea S, Jacobson KA, Salvemini D (2018). Chemotherapy-induced pain is promoted by enhanced spinal adenosine kinase levels through astrocyte-dependent mechanisms. Pain.

[31] Chen R, Yin C, Hu Q, Liu B, Tai Y, Zheng X, Li Y, Fang J, Liu B (2020). Expression profiling of spinal cord dorsal horn in a rat model of complex regional pain syndrome type-I uncovers potential mechanisms mediating pain and neuroinflammation responses. J Neuroinflammation.

[32] Khan N, Kuo A, Brockman DA, Cooper MA, Smith MT (2018). Pharmacological inhibition of the NLRP3 inflammasome as a potential target for multiple sclerosis induced central neuropathic pain. Inflammopharmacology.

[33] Huang J, Gandini MA, Chen L, M'Dahoma S, Stemkowski PL, Chung H, Muruve DA, Zamponi GW (2020). Hyperactivity of innate immunity triggers pain via TLR2-IL-33-mediated neuroimmune crosstalk. Cell Rep.

[34] He W, Long T, Pan Q, Zhang S, Zhang Y, Zhang D, Qin G, Chen L, Zhou J (2019). Microglial NLRP3 inflammasome activation mediates IL-1β release and contributes to central sensitization in a recurrent nitroglycerin-induced migraine model. J Neuroinflammation.

[35] Zhang LG, Chen J, Meng JL, Zhang Y, Liu Y, Zhan CS, Chen XG, Zhang L, Liang CZ (2019). Effect of alcohol on chronic pelvic pain and prostatic inflammation in a mouse model of experimental autoimmune prostatitis. Prostate.

[36] Widerström-Noga E (2017). Neuropathic pain and spinal cord injury: phenotypes and pharmacological management. Drugs.

[37] Donnelly CR, Chen O, Ji RR (2020). How do sensory neurons sense danger signals?. Trends Neurosci.

[38] Liu S, Mi WL, Li Q, Zhang MT, Han P, Hu S, Mao-Ying QL, Wang YQ (2015). Spinal IL-33/ST2 signaling contributes to neuropathic pain via neuronal CaMKII-CREB and astroglial JAK2-STAT3 cascades in mice. Anesthesiology.

[39] Pan Z, Shan Q, Gu P, Wang XM, Tai LW, Sun M, Luo X, Sun L (2018). Cheung CW: miRNA-23a/CXCR4 regulates neuropathic pain via directly targeting TXNIP/NLRP3 inflammasome axis. Journal of neuroinflammation.

[40] Gao F, Xiang HC, Li HP, Jia M, Pan XL, Pan HL, Li M (2018). Electroacupuncture inhibits NLRP3 inflammasome activation through CB2 receptors in inflammatory pain. Brain Behav Immun.

[41] Yoshida S, Hagiwara Y, Tsuchiya M, Shinoda M, Koide M, Hatakeyama H, Chaweewannakorn C, Suzuki K, Yano T, Sogi Y (2019). Involvement of inflammasome activation via elevation of uric acid level in nociception in a mouse model of muscle pain. Mol Pain.

[42] Xu L, Wang Q, Jiang W, Yu S, Zhang S (2019). MiR-34c ameliorates neuropathic pain by targeting NLRP3 in a mouse model of chronic constriction injury. Neuroscience.

[43] Tonkin RS, Bowles C, Perera CJ, Keating BA, Makker PGS, Duffy SS, Lees JG, Tran C, Don AS, Fath T, Liu L, O'Carroll SJ, Nicholson LFB, Green CR, Gorrie C, Moalem-Taylor G (2018). Attenuation of mechanical pain hypersensitivity by treatment with Peptide5, a connexin-43 mimetic peptide, involves inhibition of NLRP3 inflammasome in nerve-injured mice. Experimental neurology.

[44] Banach M, Juranek JK, Zygulska AL (2017). Chemotherapy-induced neuropathies-a growing problem for patients and health care providers. Brain Behav.

[45] Flatters SJL, Dougherty PM, Colvin LA (2017). Clinical and preclinical perspectives on chemotherapy-induced peripheral neuropathy (CIPN): a narrative review. Br J Anaesth.

[46] Urits I, Shen AH, Jones MR, Viswanath O, Kaye AD (2018). Complex regional pain syndrome, current concepts and treatment options. Curr Pain Headache Rep.

[47] Bruehl S (2010). An update on the pathophysiology of complex regional pain syndrome. Anesthesiology.

[48] Hu Q, Zheng X, Li X, Liu B, Yin C, Li Y, Chen R, Wang J, Liang Y, Shao X, Fang J, Liu B (2020). Electroacupuncture alleviates mechanical allodynia in a rat model of complex regional pain syndrome type-I via suppressing spinal CXCL12/CXCR4 signaling. J Pain.

[49] Coderre TJ, Xanthos DN, Francis L, Bennett GJ (2004). Chronic post-ischemia pain (CPIP): a novel animal model of complex regional pain syndrome-type I (CRPS-I; reflex sympathetic dystrophy) produced by prolonged hindpaw ischemia and reperfusion in the rat. Pain.

[50] Hu Q, Zheng X, Chen R, Liu B, Tai Y, Shao X, et al. Chronic post-ischemia pain model for complex regional pain syndrome type-I in rats. J Vis Exp. 2020;(155). 10.3791/60562. 10.3791/60562 32065161

[51] Wang J, Zheng X, Liu B, Yin C, Chen R, Li X, Li Y, Nie H, Zeng D, He X, Jiang Y, Fang J, Liu B (2020). Electroacupuncture alleviates mechanical allodynia of a rat model of CRPS-I and modulates gene expression profiles in dorsal root ganglia. Front Neurol.

[52] Yin C, Hu Q, Liu B, Tai Y, Zheng X, Li Y, Xiang X, Wang P, Liu B (2019). Transcriptome profiling of dorsal root ganglia in a rat model of complex regional pain syndrome type-I reveals potential mechanisms involved in pain. J Pain Res.

[53] Tang Y, Liu L, Xu D, Zhang W, Zhang Y, Zhou J, Huang W (2018). Interaction between astrocytic colony stimulating factor and its receptor on microglia mediates central sensitization and behavioral hypersensitivity in chronic post ischemic pain model. Brain Behav Immun.

[54] Hu Q, Wang Q, Wang C, Tai Y, Liu B, Shao X, Fang J, Liu B (2019). TRPV1 Channel contributes to the behavioral hypersensitivity in a rat model of complex regional pain syndrome type 1. Front Pharmacol.

[55] Watson JC, Sandroni P (2016). Central neuropathic pain syndromes. Mayo Clin Proc.

[56] Qian J, Zhu W, Lu M, Ni B, Yang J (2017). D-beta-hydroxybutyrate promotes functional recovery and relieves pain hypersensitivity in mice with spinal cord injury. Br J Pharmacol.

[57] Youm YH, Nguyen KY, Grant RW, Goldberg EL, Bodogai M, Kim D, D'Agostino D, Planavsky N, Lupfer C, Kanneganti TD, Kang S, Horvath TL, Fahmy TM, Crawford PA, Biragyn A, Alnemri E, Dixit VD (2015). The ketone metabolite beta-hydroxybutyrate blocks NLRP3 inflammasome-mediated inflammatory disease. Nat Med.

[58] Liu BY (2009). Zhang HL: [Bradykinin modulates ion channel in inflammatory pain]. Yao Xue Xue Bao.

[59] Liu B, Linley JE, Du X, Zhang X, Ooi L, Zhang H, Gamper N (2010). The acute nociceptive signals induced by bradykinin in rat sensory neurons are mediated by inhibition of M-type K+ channels and activation of Ca2 + -activated Cl- channels. J Clin Invest.

[60] Zheng X, Tai Y, He D, Liu B, Wang C, Shao X, Jordt SE, Liu B (2019). ETAR and protein kinase A pathway mediate ET-1 sensitization of TRPA1 channel: a molecular mechanism of ET-1-induced mechanical hyperalgesia. Mol Pain.

[61] Calil IL, Zarpelon AC, Guerrero AT, Alves-Filho JC, Ferreira SH, Cunha FQ, Cunha TM, Verri WA (2014). Lipopolysaccharide induces inflammatory hyperalgesia triggering a TLR4/MyD88-dependent cytokine cascade in the mice paw. PLoS One.

[62] Lieberthal J, Sambamurthy N, Scanzello CR (2015). Inflammation in joint injury and post-traumatic osteoarthritis. Osteoarthritis Cartilage.

[63] Yu ML, Wei RD, Zhang T, Wang JM, Cheng Y, Qin FF, Fu SP, Lu ZG, Lu SF (2020). Electroacupuncture relieves pain and attenuates inflammation progression through inducing IL-10 production in CFA-induced mice. Inflammation.

[64] Yu S, Zhao G, Han F, Liang W, Jiao Y, Li Z, Li L (2020). Muscone relieves inflammatory pain by inhibiting microglial activation-mediated inflammatory response via abrogation of the NOX4/JAK2-STAT3 pathway and NLRP3 inflammasome. Int Immunopharmacol.

[65] Fattori V, Hohmann MSN, Rossaneis AC, Manchope MF, Alves-Filho JC, Cunha TM, Cunha FQ, Verri WA (2017). Targeting IL-33/ST2 signaling: regulation of immune function and analgesia. Expert Opin Ther Targets.

[66] Yin C, Liu B, Li Y, Li X, Wang J, Chen R, Tai Y, Shou Q, Wang P, Shao X, Liang Y, Zhou H, Mi W, Fang J, Liu B (2020). IL-33/ST2 induces neutrophil-dependent reactive oxygen species production and mediates gout pain. Theranostics.

[67] Rees F, Hui M, Doherty M (2014). Optimizing current treatment of gout. Nat Rev Rheumatol.

[68] Safiri S, Kolahi AA, Cross M, Carson-Chahhoud K, Hoy D, Almasi-Hashiani A, et al. Prevalence, incidence, and years lived with disability due to gout and its attributable risk factors for 195 countries and territories 1990-2017: a systematic analysis of the global burden of disease study 2017. Arthritis Rheumatol. 2020. Online ahead of print. 10.1002/art.41404 32755051

[69] Szekanecz Z, Szamosi S, Kovacs GE, Kocsis E, Benko S (2019). The NLRP3 inflammasome - interleukin 1 pathway as a therapeutic target in gout. Arch Biochem Biophys.

[70] Mariotte A, De Cauwer A, Po C, Abou-Faycal C, Pichot A, Paul N, Aouadi I, Carapito R, Frisch B, Macquin C (2020). A mouse model of MSU-induced acute inflammation in vivo suggests imiquimod-dependent targeting of Il-1beta as relevant therapy for gout patients. Theranostics.

[71] Chen CJ, Shi Y, Hearn A, Fitzgerald K, Golenbock D, Reed G, Akira S, Rock KL (2006). MyD88-dependent IL-1 receptor signaling is essential for gouty inflammation stimulated by monosodium urate crystals. J Clin Invest.

[72] So A, De Smedt T, Revaz S, Tschopp J (2007). A pilot study of IL-1 inhibition by anakinra in acute gout. Arthritis Res Ther.

[73] Torres R, Macdonald L, Croll SD, Reinhardt J, Dore A, Stevens S, Hylton DM, Rudge JS, Liu-Bryan R, Terkeltaub RA, Yancopoulos GD, Murphy AJ (2009). Hyperalgesia, synovitis and multiple biomarkers of inflammation are suppressed by interleukin 1 inhibition in a novel animal model of gouty arthritis. Ann Rheum Dis.

[74] Amaral FA, Costa VV, Tavares LD, Sachs D, Coelho FM, Fagundes CT, Soriani FM, Silveira TN, Cunha LD, Zamboni DS, Quesniaux V, Peres RS, Cunha TM, Cunha FQ, Ryffel B, Souza DG, Teixeira MM (2012). NLRP3 inflammasome-mediated neutrophil recruitment and hypernociception depend on leukotriene B(4) in a murine model of gout. Arthritis Rheum.

[75] Yin C, Liu B, Wang P, Li X, Li Y, Zheng X, et al. Eucalyptol alleviates inflammation and pain responses in a mouse model of gout arthritis. Br J Pharmacol. 2020;177(9):2042–57. 10.1111/bph.14967. 10.1111/bph.14967 PMC7161556 31883118

[76] Trevisan G, Hoffmeister C, Rossato MF, Oliveira SM, Silva MA, Silva CR, Fusi C, Tonello R, Minocci D, Guerra GP, Materazzi S, Nassini R, Geppetti P, Ferreira J (2014). TRPA1 receptor stimulation by hydrogen peroxide is critical to trigger hyperalgesia and inflammation in a model of acute gout. Free Radic Biol Med.

[77] Trevisan G, Hoffmeister C, Rossato MF, Oliveira SM, Silva MA, Ineu RP, Guerra GP, Materazzi S, Fusi C, Nassini R, Geppetti P, Ferreira J (2013). Transient receptor potential ankyrin 1 receptor stimulation by hydrogen peroxide is critical to trigger pain during monosodium urate-induced inflammation in rodents. Arthritis Rheum.

[78] Tschopp J, Schroder K (2010). NLRP3 inflammasome activation: The convergence of multiple signalling pathways on ROS production?. Nat Rev Immunol.

[79] Zhou R, Tardivel A, Thorens B, Choi I, Tschopp J (2010). Thioredoxin-interacting protein links oxidative stress to inflammasome activation. Nat Immunol.

[80] Chapman CR, Vierck CJ (2017). The transition of acute postoperative pain to chronic pain: an integrative overview of research on mechanisms. J Pain.

[81] Ghasemlou N, Chiu IM, Julien JP, Woolf CJ (2015). CD11b + Ly6G- myeloid cells mediate mechanical inflammatory pain hypersensitivity. Proc Natl Acad Sci U S A.

[82] Cowie AM, Menzel AD, O'Hara C, Lawlor MW, Stucky CL (2019). NOD-like receptor protein 3 inflammasome drives postoperative mechanical pain in a sex-dependent manner. Pain.

[83] Wolf G, Livshits D, Beilin B, Yirmiya R, Shavit Y (2008). Interleukin-1 signaling is required for induction and maintenance of postoperative incisional pain: genetic and pharmacological studies in mice. Brain Behav Immun.

[84] Hu Y, Liang D, Li X, Liu HH, Zhang X, Zheng M, Dill D, Shi X, Qiao Y, Yeomans D, Carvalho B, Angst MS, Clark JD, Peltz G (2010). The role of interleukin-1 in wound biology. Part I: Murine in silico and in vitro experimental analysis. Anesth Analg.

[85] Hu Y, Liang D, Li X, Liu HH, Zhang X, Zheng M, Dill D, Shi X, Qiao Y, Yeomans D, Carvalho B, Angst MS, Clark JD, Peltz G (2010). The role of interleukin-1 in wound biology. Part II: In vivo and human translational studies. Anesth Analg.

[86] Rohde C, Chiang A, Adipoju O, Casper D, Pilla AA (2010). Effects of pulsed electromagnetic fields on interleukin-1 beta and postoperative pain: a double-blind, placebo-controlled, pilot study in breast reduction patients. Plast Reconstr Surg.

[87] Cowie AM, Dittel BN, Stucky CL (2019). A novel sex-dependent target for the treatment of postoperative pain: the NLRP3 inflammasome. Front Neurol.

[88] Yoshida S, Hagiwara Y, Tsuchiya M, Shinoda M, Koide M, Hatakeyama H, Chaweewannakorn C, Yano T, Sogi Y, Itaya N (2018). Involvement of neutrophils and interleukin-18 in nociception in a mouse model of muscle pain. Mol Pain.

[89] Sarzi-Puttini P, Giorgi V, Marotto D, Atzeni F (2020). Fibromyalgia: an update on clinical characteristics, aetiopathogenesis and treatment. Nat Rev Rheumatol.

[90] Littlejohn G (2015). Neurogenic neuroinflammation in fibromyalgia and complex regional pain syndrome. Nat Rev Rheumatol.

[91] Balsom PD, Seger JY, Sjodin B, Ekblom B (1992). Physiological responses to maximal intensity intermittent exercise. Eur J Appl Physiol Occup Physiol.

[92] Goadsby PJ, Holland PR (2019). An update: pathophysiology of migraine. Neurol Clin.

[93] May A, Schulte LH (2016). Chronic migraine: risk factors, mechanisms and treatment. Nat Rev Neurol.

[94] Yucel M, Kotan D, Gurol Ciftci G, Ciftci IH, Cikriklar HI (2016). Serum levels of endocan, claudin-5 and cytokines in migraine. Eur Rev Med Pharmacol Sci.

[95] Han D (2019). Association of serum levels of calcitonin gene-related peptide and cytokines during migraine attacks. Ann Indian Acad Neurol.

[96] de Vries T, Villalon CM, MaassenVanDenBrink A (2020). Pharmacological treatment of migraine: CGRP and 5-HT beyond the triptans. Pharmacol Ther.

[97] Marone IM, De Logu F, Nassini R, De Carvalho GM, Benemei S, Ferreira J, Jain P, Li Puma S, Bunnett NW, Geppetti P, Materazzi S (2018). TRPA1/NOX in the soma of trigeminal ganglion neurons mediates migraine-related pain of glyceryl trinitrate in mice. Brain.

[98] Haanes KA, Edvinsson L (2019). Pathophysiological mechanisms in migraine and the identification of new therapeutic targets. CNS Drugs.

[99] Liu Y, Mikrani R, Xie D, Wazir J, Shrestha S, Ullah R, Baig M, Ahmed A, Srivastava PK, Thapa KB, Zhou X (2020). Chronic prostatitis/chronic pelvic pain syndrome and prostate cancer: study of immune cells and cytokines. Fundam Clin Pharmacol.

[100] Polackwich AS, Shoskes DA (2016). Chronic prostatitis/chronic pelvic pain syndrome: a review of evaluation and therapy. Prostate Cancer Prostatic Dis.

[101] Zhang Z, Li Z, Yu Q, Wu C, Lu Z, Zhu F, Zhang H, Liao M, Li T, Chen W, Xian X, Tan A, Mo Z (2015). The prevalence of and risk factors for prostatitis-like symptoms and its relation to erectile dysfunction in Chinese men. Andrology.

[102] Falk S, Dickenson AH (2014). Pain and nociception: mechanisms of cancer-induced bone pain. J Clin Oncol.

[103] Kane CM, Hoskin P, Bennett MI (2015). Cancer induced bone pain. BMJ.

[104] Srikrishna G, Freeze HH (2009). Endogenous damage-associated molecular pattern molecules at the crossroads of inflammation and cancer. Neoplasia.

[105] Volkow ND, McLellan AT (2016). Opioid abuse in chronic pain--misconceptions and mitigation strategies. N Engl J Med.

[106] Cai Y, Kong H, Pan YB, Jiang L, Pan XX, Hu L, Qian YN, Jiang CY, Liu WT (2016). Procyanidins alleviates morphine tolerance by inhibiting activation of NLRP3 inflammasome in microglia. J Neuroinflammation.

[107] Wang H, Zhang Y, Ma X, Wang W, Xu X, Huang M, Xu L, Shi H, Yuan T, Jiang W, Wang A, Xu T (2020). Spinal TLR4/P2X7 receptor-dependent NLRP3 inflammasome activation contributes to the development of tolerance to morphine-induced antinociception. J Inflamm Res.

[108] Grace PM, Strand KA, Galer EL, Rice KC, Maier SF, Watkins LR (2018). Protraction of neuropathic pain by morphine is mediated by spinal damage associated molecular patterns (DAMPs) in male rats. Brain Behav Immun.

